# pyAmpli: an amplicon-based variant filter pipeline for targeted resequencing data

**DOI:** 10.1186/s12859-017-1985-1

**Published:** 2017-12-14

**Authors:** Matthias Beyens, Nele Boeckx, Guy Van Camp, Ken Op de Beeck, Geert Vandeweyer

**Affiliations:** 1 Center of Medical Genetics, University of Antwerp, Prins Boudewijnlaan 43, 2650 Antwerp, Belgium; 2 Center of Oncological Research, University of Antwerp, Universiteitsplein 1, 2610 Antwerp, Belgium

**Keywords:** Targeted resequencing, Variant filtering, Somatic, Germline, Next-generation sequencing

## Abstract

**Background:**

Haloplex targeted resequencing is a popular method to analyze both germline and somatic variants in gene panels. However, involved wet-lab procedures may introduce false positives that need to be considered in subsequent data-analysis. No variant filtering rationale addressing amplicon enrichment related systematic errors, in the form of an all-in-one package, exists to our knowledge.

**Results:**

We present pyAmpli, a platform independent parallelized Python package that implements an amplicon-based germline and somatic variant filtering strategy for Haloplex data. pyAmpli can filter variants for systematic errors by user pre-defined criteria. We show that pyAmpli significantly increases specificity, without reducing sensitivity, essential for reporting true positive clinical relevant mutations in gene panel data.

**Conclusions:**

pyAmpli is an easy-to-use software tool which increases the true positive variant call rate in targeted resequencing data. It specifically reduces errors related to PCR-based enrichment of targeted regions.

**Electronic supplementary material:**

The online version of this article (10.1186/s12859-017-1985-1) contains supplementary material, which is available to authorized users.

## Background

Low-cost targeted resequencing using specific gene panels in large sample cohorts is widely used in diagnostic settings and forms the current gold standard for multiple reasons. For instance, in hearing loss, screening of specific genes can be more efficient than whole exome, or whole genome sequencing due to reduced sequencing and analysis costs [1]. Second, data interpretation outside known disease genes is difficult and has limited added value in clinical settings. Finally, it is a cost-effective technique for ultra-deep sequencing which enables detection of low-allelic variants, for instance needed to pinpoint subclonal IgHV rearrangements in chronic lymphocytic leukemia [2].

**Fig. 1:**
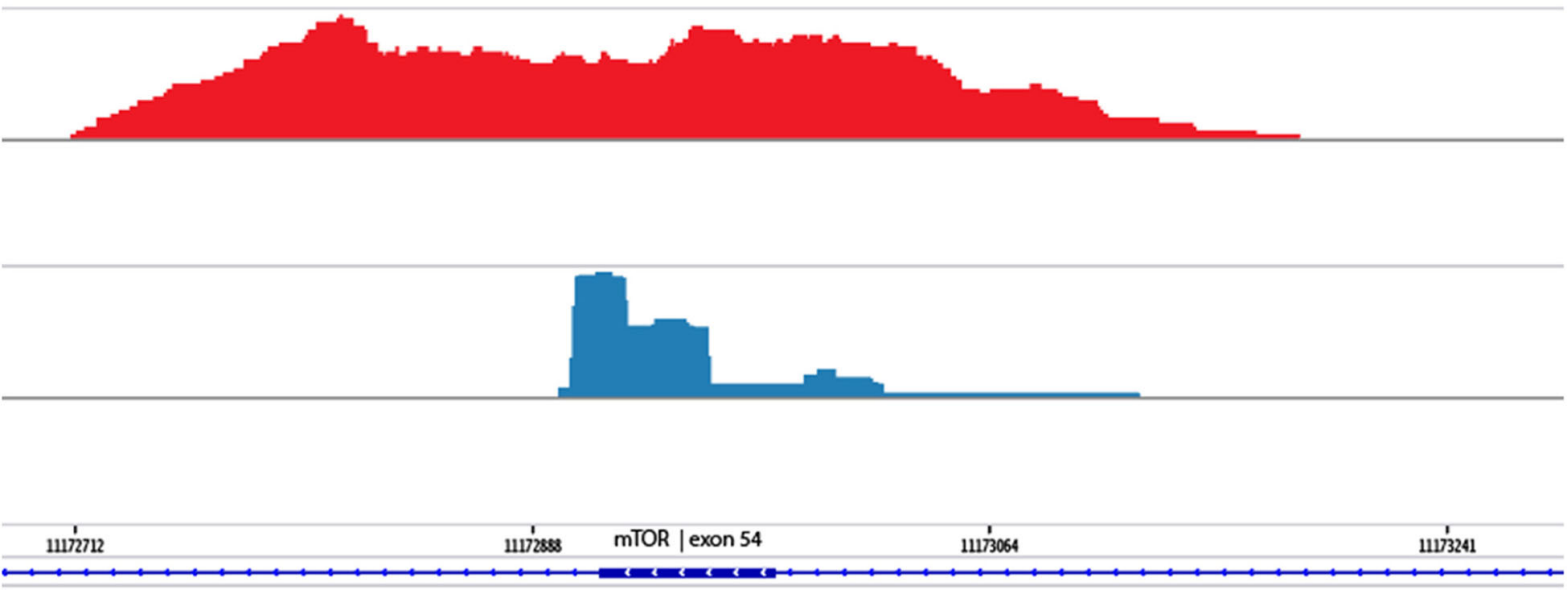
Sashimi target enrichment plots. *mTOR* Exon 54 coverage for two different target enrichment methods is represented by a Sashimi plot: 1) hybridization capture-based technique, showing more bell-shaped enrichment of random fragments (red histogram) and 2) amplicon- or multiplex PCR-based approach, showing vertical enrichment blocks of identical fragments (blue histogram)

**Fig. 2:**
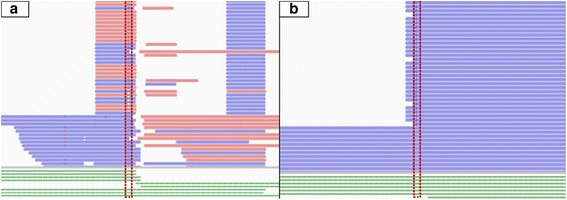
Vertical read blocks and variant calling bias visualization. Typical vertically enriched read blocks are illustrated in (2**a** and 2**b**). Aspecific fragments, not corresponding to predicted amplicons are shown in (2**a)** and variants restricted to read ends are shown in (2**b**). Called variants are indicated by a red dashed rectangular. Reads are given in blue and pink colored horizontal bars, indicating read orientation. Theoretical manufacturer designed Haloplex probes are presented in green colored horizontal bars below their corresponding enriched reads

Target enrichment methods can be divided into amplicon or multiplex PCR-based approaches, showing vertical enrichment blocks of identical fragments, and hybridization capture-based techniques, showing more bell-shaped enrichment of random fragments (Fig. 1) [3]. Here, we focused specifically on the analysis of the Haloplex Target Enrichment System, which can enrich up to thousands of exons. The Haloplex technology was originally developed by Olink Bioscience (prof. Olle Ericsson, Uppsala, Sweden) from where it has been commercialized by the spin-off company Halo Genomics. To date, the technology is further developed and supplied by Agilent Technologies (Santa Clara, USA). Although the technique is hybridization based, it results in amplicon-like data due to non-random restriction enzyme fragmentation and subsequent PCR amplification. The ligation-dependent selection for circular fragments increases target specificity towards fragments where the start and end positions correspond to restriction sites. However, a significant fraction of aspecific amplicons, not corresponding to predicted restriction fragments, is often present in the library, and can induce spurious variants. These variants can be visually recognised by not being present in genuine amplicons (Fig. 2a). Second, coverage is not uniform across the captured fragments, possibly resulting in false-negative heterozygous variants when both alleles are not sufficiently captured. Finally, PCR duplicates cannot be removed without the usage of molecular barcode tags, as these are inherent to the technology. Here, one could hypothesize that true variants should be present in multiple overlapping amplicons, as these correspond to independent captures by definition [4]. Introduction of incorrect nucleotides during PCR is therefore indistinguishable from true subclonal variants with low-allelic frequency.

To the best of our knowledge, no variant filtering rationale, in the format of an all-in-one package, exists that takes these targeted resequencing specific biases into account to differentiate false-positive from true-positive variants. Here, we present pyAmpli, a platform independent parallelized Python package that leverages amplicon specific information during variant filtering. Although user applied variant calling algorithms (e.g. VarScan2 and GATK Unified Genotyper) return various variant quality and reliability scores, these parameters are limited in amplicon- or PCR-based enrichment methods as they do not include amplicon information. Further, they are only suitable for hard filtering of variants. As such, variant hard filtering is exclusively based on the information available in the variant calling file generated by the chosen variant caller algorithm. pyAmpli uses solely the variant calling file for extraction of the variant’s position and further uses the sample’s alignment file to extract amplicon information.

pyAmpli can be applied in an oncological setting, after somatic tumor-normal variant calling as well in germline disease-gene screening projects. Our variant filtering algorithm ensures an enrichment of true-positive variants via a seven-step multi-staged categorization pipeline (Additional file 1).

## Implementation

The pyAmpli package is developed using Python 2.7 [5]. The package is freely available for downstream variant analysis across various computing platforms. pyAmpli requires the following dependencies: *pysam* [6] is required for reading alignment files, *PyVCF* [7] for reading, formatting and generating variant files and *pyYAML* [8] for reading the user configuration file.

### Input

The package provides both somatic and germline variant filtering. The default somatic mode of pyAmpli requires an amplicon-design file provided by the manufacturer, a paired tumor-normal variant calling file (VCF) and a normal and tumor alignment file (BAM) as input. The amplicon-design file is a BED file containing genomic location and probe identifier names for each included restriction fragment. Germline variants filtering requires an amplicon-design file, a single sample VCF and alignment file. Default and optimized settings for both somatic and germline parameters are included in the software as YAML configuration files. Command-line usage of pyAmpli in somatic mode is illustrated by Listing 1.

pyAmpli **.** py somatic

- bn *normal_sample_chr1.bam.*
- bt *tumor_sample_chr1.bam.*
- v *somatic_variants_chr1.vcf.*
- d *amplicon_design_chr1.bed.*
- od *output_directory*

Listing 1

### Variant processing

The variant processing workflow of pyAmpli can be summarized as follows. If supplied, a configuration file with user-defined thresholds is read-in, otherwise default settings are used. Next, the amplicon-design file provided by the manufacturer is processed into an easy accessible dictionary. Subsequently, every input variant present in the VCF is subjected to variant filtering analysis. The main variant analysis starts by assigning aligned read pairs overlapping the variant position to amplicons specified in the design file, discarding aspecific amplicons. Second, the ratio of variant-containing amplicons over all predicted amplicons covering the target position is calculated. Based on this ratio, variants are then categorized in 7 categories, as discussed below: *DepthFail*, *OneAmpPass*, *LowAmpFail*, *MatchAmpPass*, *PositionFail*, *NormalFail* and *AmpPass*. pyAmpli adds the final variant category to the *FILTER* field of the output VCF file (v4.1-formatted). Additional metrics, including the amplicon ratio and several amplicon counts are added to the *INFO* field to allow users to easily perform further downstream selection of variant categories (Table 1). A detailed decision diagram of pyAmpli’s filter logic is given in Additional file 1.

**Table 1:** Additional VCF information fields. After running pyAmpli a new VCF is generated with additional *INFO* fields. These fields provide the user information on amplicon fractions, counts and offsets of reference and alternative alleles

INFO ID	Description
AmpFR	Amplicon fraction for reference allele
AmpFA	Amplicon fraction for alternative allele
AmpCR	Amplicon count for reference allele
AmpCA	Amplicon count for alternative allele
AmpC	Amplicon total count
AmpF_OA	Amplicon count offset compared to allelic depth, for alternative allele
AmpF_OR	Amplicon count offset compared to allelic depth, for reference allele

### Variant categories

Variants are evaluated for each of the following criteria, in the order given here, and assigned to the first matching category (Additional file 1). When all criteria are passed, a variant is classified as high quality, corresponding to the label *AmpPass.*

#### DepthFail: variants with low read evidence

In a first step, variants with insufficient coverage by genuine read-pairs are flagged as low read evidence variants, and not subjected to further variant filtering. *FILTER* field flags *DepthFail* and *DepthFailTumor/Normal* are set, respectively in germline and somatic modes. Users have the flexibility to define their own *DepthFail* cut-off by adjusting the *min_depth_normal* and/or *min_depth_tumor* values in the configuration file.

#### OneAmpPass: variants with panel design limitations

Variants covered by and present in a single theoretical amplicon, as a design limitation, might be more prone for systemic enrichment artefacts. As we have insufficient information to evaluate the reliability of these variants, they are flagged as *OneAmpPass,* and not subjected to further filtering.

#### LowAmpFail: variants with low amount of covered amplicons

When variants are covered by multiple theoretical amplicons, we can infer variant reliability based on the number of amplicons containing the variant. Variants covered by more than two overlapping theoretical amplicons are flagged as *LowAmpFail* if the alternative allele is present in reads corresponding to less than three of these amplicons.

Variants covered by just two theoretical amplicons are handled separately. These variants are flagged as *LowAmpFail* if the alternative allele is present in reads corresponding to only one of both amplicons.

#### MatchAmpPass: variants with low amount of covered amplicons

Variants covered by just two theoretical amplicons are handled separately as *MatchAmpPass* if the alternative allele is present in reads from both amplicons, to indicate the limited discriminative power.

#### PositionFail: positional biases

Variants only present in the first two positions of either 3′ or 5′ read ends are flagged as *PositionFail*. This enrichment artefact is typically seen in Haloplex gene panels, because fragments are reproducibly generated by restriction enzymes, which cut only recognized sequences and generate non-random fragments [9]. Users can adjust the *min_read_pos* (default 2) and *min_read_pos_fraction* (default 10) in the configuration file, i.e. variants will be flagged as *PositionFail* if more than 10% of the total reads contain the alternative allele in the first two positions of either 3′ or 5′ read ends.

#### NormalFail: low-fraction variants in normal samples

This filter is only applied in somatic mode and is more subjective to user settings. When considering paired tumor-normal samples, somatic variants are not expected to be present in the patient’s paired normal tissue sample. First, this can be indicative for a false-positive somatic variant in the tumor tissue sample, that is in fact a true-positive low-fraction germline variant in the normal sample. Secondly, it might be a systemic enrichment artefact that is more pronounced in the tumor sample and therefore called as somatic. Lastly, it could be a reliable somatic variant. This may be explained by field cancerization, which is the occurrence of genetic, epigenetic and biochemical aberrations in structurally intact cells in histologically normal tissue adjacent to cancerous lesions [10]. By default, somatic variants present in more than 1% of reads from the normal sample are flagged as *NormalFail*. To allow the effect of field cancerization, the user can adjust the threshold (*min_frac*) for flagging these variants in the configuration file.

#### AmpPass: threshold-passing variants

As mentioned above, variants passing all user-defined filters are flagged as high-quality variants, using the *AmpPass* label.

### Performance evaluation

We benchmarked pyAmpli on VCF and BAM files generated on in-house data. We calculated and validated the true and false positive rates. Next, we estimated runtime for batch processing.

### Pre-pyAmpli bioinformatic processing of benchmark samples

Haloplex libraries were generated following the manufacturers guidelines (Protocol F1, July 2015, Agilent, CA, USA) and sequenced on an Illumina HiSeq1500 platform. Reads were trimmed for adapter sequence with Trimmomatic *v0.36*, and aligned with BWA *v0.7.4* to version hg19 of the human genome. Germline variants were called using GATK Unified Genotyper *v3.3.0* on 21 normal colon tissue samples. Somatic/loss-of-heterozygosity (LOH) variants were called using VarScan2 *v2.3.9* on 115 colon tumor-normal tissue pairs. Tumor sample is defined as either primary colon tumor or metastatic tissue.

### Benchmarking

True and false positive rates were estimated as follows. Variants present in ExAc *r1.0*, COSMIC *v81* or dbSNP *v142* databases were assumed true positive, and false positive otherwise. Next, variants were categorized according to variant type (germline, somatic and LOH) and filtering status (i.e. passing pyAmpli filtering or failing). To validate pyAmpli variant classification, 37 somatic variants were selected and validated by Sanger sequencing on a 3130xl Genetic Analyzer platform (Applied Biosystems Inc.).

## Results and discussion

Current variant calling algorithms return variant quality and reliability scores in their VCFs. The calculated scores do not provide any amplicon information for reliable variant filtering. Further, necessity for amplicon-based filtering was made clear in a Sanger sequencing validation experiment by Samorodnitsky and colleagues. They showed that alternative alleles covered by less amplicons than present in their design are prone to be false positive findings [9].

There are analysis pipelines optimized for amplicon sequencing data, such as SureCall (Agilent Technologies, USA) and SeqNext (JSI medical systems, Germany), available. Although, latter software packages are able to call variants, the downstream variant filtering relies on ‘hard’ filters and information regarding the amplicon itself is lacking. Further, researchers still need to visually inspect all the data and judge the validity, and eventually manually flag the variants, which is a time-consuming step. Another disadvantage of these tools is that they are incompatible for paired variant calling. Of course, we do not discourage using SureCall or SeqNext variant analysis pipeline. The software output can serve as input for pyAmpli. To the best of our knowledge, no downstream post-processing tools as pyAmpli exists. pyAmpli will add useful variant and amplicon parameters that will guide the end-user for a legit variant interpretation and a hopefully desired decrease in analysis time per patient.

We present a new convenient variant filtering tool pyAmpli targeted at the reduction of systematic biases present in resequencing data generated using amplicon-based enrichment protocols. These protocols give rise to recurrent artefacts, as illustrated for Haloplex enrichment in Fig. 2. First, aspecifically enriched amplicons can introduce false positive variant calls. In case of Haloplex, these can be identified by the absence of corresponding restriction sites in the design file. Consequently, variants present only in aspecific amplicons and absent from genuine amplicons, can be labelled as false positives (*LowAmpFail* category, Fig. 2a). Second, Fig. 2b shows variants restricted to read ends, likely corresponding to systemic enrichment artefacts (*PositionFail* category). Whereas these artefacts are relevant for both germline and somatic variant evaluation purposes, an additional filter is present to specifically evaluate somatic variants.

### pyAmpli true and false positive rate

In general, applying the pyAmpli germline filter on GATK Unified Genotyper calls of 21 normal tissue samples, increases the true positive rate from 39% (15,673 variants) to 64% (11,368) (Table 2). VarScan2 is proven to be a sensitive caller, however the tumor-normal variant calls lack high specificity. Applying pyAmpli somatic filtering settings on somatic and LOH variants of 115 tumor-normal tissue pairs increases the true positive rate from 29% (4028) and 45% (934) to 37% (885) and 81% (208), respectively (Table 2). After validation by Sanger sequencing of 37 variants, 21, 12, 4 and 0 variants were categorized as true positive, true negative, false positive and false negative, respectively (Additional file 2).

**Table 2:** pyAmpli true and false positive rates.False and true positive rates in percentages before (−) and after (+) pyAmpli germline, somatic and LOH variant filtering with corresponding total variant number for ratio calculation. Germline variants were called with the GATK Unified Genotyper. Somatic and LOH variants were called with VarScan2

Variant	Filter	True positive rate (%)	False positive rate (%)	Number of variants
Germline	–	39	61	15,673
	+	64	36	11,368
Somatic	–	29	71	4028
	+	37	63	885
LOH	–	45	55	934
	+	81	19	208

pyAmpli allows the user to select for true positive variants in gene panel data. Further, user-defined settings, based on their in-house validation cohorts, can be implemented in the variant filtering by adjusting the YAML configuration file.

### Performance

We obtained the time required for variant filtering using a set of 115 colon tumor-normal pairs with an average of 289 variants per sample. Using the available parallel variant filtering functionality with 16 processing threads, we obtained an average CPU runtime per variant of 16.03 ms (16-core AMD Opteron™ 6378, 64-bit Linux 4.4.0–22-generic) (Additional file 3). Further upscaling has marginal benefits due to I/O limitations.

## Conclusions

pyAmpli is a fast and parallel python program tailored to improve moderate true positive rates and reduce high false positive rates observed in PCR-based targeted enrichment strategies, in comparison to hybridisation-based capturing approaches. Although it was validated on Haloplex data, its principles are applicable to all PCR-based methods, such as Molecular Inversion Probes (MIPs) or multiplex PCR. Usage requires minimal input and limited programming skills from the user and only commodity computational resources. Output is generated in VCF v4.1 format and can be easily post-processed by the user.

## Availability and requirements

**Project name**: pyAmpli**.**

**Project home page**: https://mbeyens.github.io/pyAmpli. The repository provides the package, quick-start examples and command-line examples for easy testing and performing essential processing.

**Operating system(s)**: any supporting Python 2.7 (tested on Ubuntu 14.04.4 LTS)**.**

**Programming language**: Python 2.7**.**

**Other requirements**: pysam > =0.8.4, PyVCF > =0.6.8, pyYAML > =3.11, setuptools > =20.2.2, samtools > =0.1.18, pigz > =2.3.4**.**

**License**: The GPL-v3 license (https://opensource.org/licenses/GPL-3.0).

**Any restrictions to use by non-academics**: None

## Additional files

Additional file 1: pyAmpli variant filter decision diagram. (DOCX 209 kb)

Additional file 2: Sanger sequencing variant validation. (DOCX 71 kb)

Additional file 3: pyAmpli CPU runtime. (DOCX 132 kb)

## Abbreviations

BAM: Binary alignment file
LOH: Loss-of-heterozygosity
MIPs: Molecular inversion probes
PCR: Polymerase chain reaction
VCF: Variant calling file
