# Supplementary material for: pyAmpli: an amplicon-based variant filter pipeline for targeted resequencing data
Source: BMC Bioinformatics. 2017 Dec 14;18:554. doi: 10.1186/s12859-017-1985-1 (PMC5729461; doi:10.1186/s12859-017-1985-1)
Supplement: Supplementary file 1 — pyAmpli variant filter decision diagram. (DOCX 209 kb) [file 12859_2017_1985_MOESM1_ESM.docx]

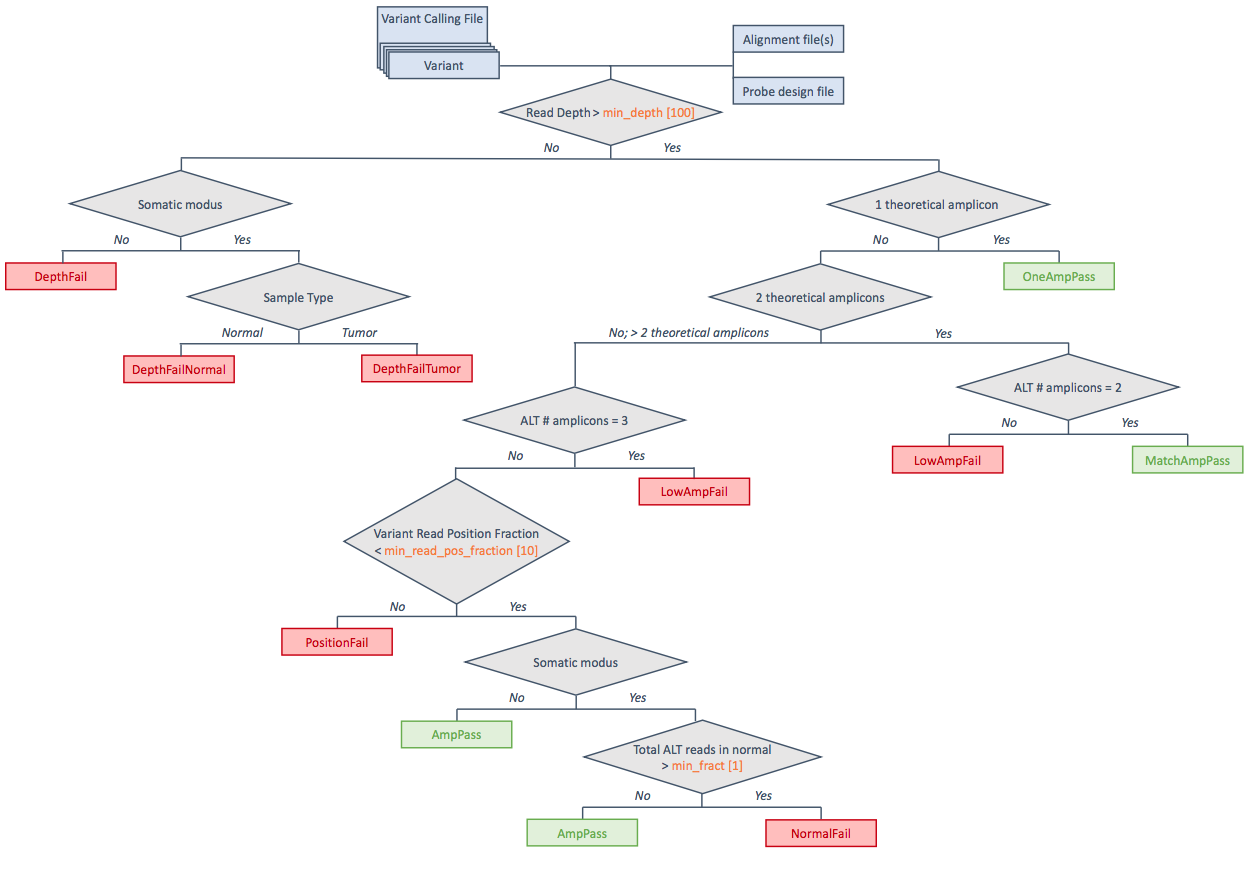


Supplementary material A – pyAmpli variant filter decision diagram

The filter logic behind pyAmpli tool. Variant calling file, alignment file and probe design file are given as input. Orange colored text is user-specific [default]. Variants passing the criteria are given the green colored filter flag. Variants failing a specific filter step receive the red colored flag.
