# Supplementary material for: pyAmpli: an amplicon-based variant filter pipeline for targeted resequencing data
Source: BMC Bioinformatics. 2017 Dec 14;18:554. doi: 10.1186/s12859-017-1985-1 (PMC5729461; doi:10.1186/s12859-017-1985-1)
Supplement: Supplementary file 2 — Sanger sequencing variant validation. (DOCX 71 kb) [file 12859_2017_1985_MOESM2_ESM.docx]

| **Chromosome** | **Position** | **Reference allele** | **Alternative allele** | | **Sanger nucleotide** | **pyAmpli filter** | **Validation label** |
| --- | --- | --- | --- | --- | --- | --- | --- |
| 1 | 27106272 | T | A | A | | AmpPass | TP |
| 2 | 49216277 | C | T | T | | MatchAmpPass | TP |
| 3 | 12645699 | G | C | C | | AmpPass | TP |
| 3 | 12645701 | T | A | A | | AmpPass | TP |
| 4 | 177608950 | G | A | G | | AmpPass | FP |
| 4 | 1803274 | C | T | T | | AmpPass | TP |
| 5 | 112173917 | C | T | T | | AmpPass | TP |
| 5 | 112174929 | C | G | G | | MatchAmpPass | TP |
| 5 | 112175174 | G | T | T | | AmpPass | TP |
| 5 | 112175303 | C | T | T | | AmpPass | TP |
| 5 | 112175466 | C | A | A | | AmpPass | TP |
| 5 | 112175761 | T | G | T | | LowAmpFail | TN |
| 5 | 112175778 | C | G | C | | PositionFail | TN |
| 6 | 43752377 | C | T | C | | MatchAmpPass | FP |
| 7 | 140453136 | A | T | T | | AmpPass | TP |
| 7 | 55224484 | C | T | C | | MatchAmpPass | FP |
| 7 | 55269427 | G | A | A | | AmpPass | TP |
| 8 | 38285400 | G | C | G | | LowAmpFail | TN |
| 9 | 101904938 | C | T | C | | LowAmpFail | TN |
| 10 | 89692827 | T | C | C | | AmpPass | TP |
| 11 | 108186629 | C | T | C | | LowAmpFail | TN |
| 11 | 108187232 | G | A | G | | LowAmpFail | TN |
| 11 | 108187246 | C | T | C | | LowAmpFail | TN |
| 12 | 25398279 | A | T | A | | NormalFail | TN |
| 12 | 25398284 | C | A | A | | MatchAmpPass | TP |
| 12 | 25398555 | C | T | T | | AmpPass | TP |
| 14 | 105242910 | G | A | G | | MatchAmpPass | FP |
| 14 | 75747476 | T | G | T | | LowAmpFail | TN |
| 14 | 75747496 | C | G | C | | PositionFail | TN |
| 17 | 7576022 | G | A | A | | AmpPass | TP |
| 17 | 7577094 | G | A | A | | AmpPass | TP |
| 17 | 7577544 | A | G | G | | AmpPass | TP |
| 17 | 7578263 | G | A | A | | AmpPass | TP |
| 17 | 7578406 | C | T | T | | MatchAmpPass | TP |
| 17 | 7578411 | C | T | T | | AmpPass | TP |
| 18 | 45395575 | T | C | T | | PositionFail | TN |
| 19 | 45924427 | C | A | C | | LowAmpFail | TN |

Supplementary material B – Sanger sequencing variant validation

After Sanger sequencing validation of 37 variants, 21, 12, 4 and 0 variants were categorized as true positive (TP), true negative (TN), false positive (FP) and false negative (FN), respectively.
