# Supplementary material for: pyAmpli: an amplicon-based variant filter pipeline for targeted resequencing data
Source: BMC Bioinformatics. 2017 Dec 14;18:554. doi: 10.1186/s12859-017-1985-1 (PMC5729461; doi:10.1186/s12859-017-1985-1)
Supplement: Supplementary file 3 — pyAmpli CPU runtime. (DOCX 132 kb) [file 12859_2017_1985_MOESM3_ESM.docx]

**Supplementary material C – pyAmpli CPU runtime**

Number of cores versus CPU runtime per sample in seconds. Sample A, B, C and D contain 336, 470, 807 and 163 variants respectively. A gradual decrease in CPU runtime is seen in the figure. Usage of more than 16 cores has a limited positive runtime effect. The average CPU runtime per variant filter on a 16-core AMD Opteron™ 6378 processor (64-bit Linux 4.4.0-22-generic) and a 32-core processor is 16.03 ms and 15.58 ms, respectively. This marginal gain in time can be assigned to I/O handling.
